# Chromosome congression explained by nanoscale electrostatics

**DOI:** 10.1186/1742-4682-11-12

**Published:** 2014-02-24

**Authors:** L John Gagliardi, Daniel H Shain

**Affiliations:** 1 Department of Physics, Rutgers The State University of New Jersey, Camden, NJ 08102, USA; 2 Department of Biology, Rutgers The State University of New Jersey, Camden, NJ 08102, USA

## Abstract

Nanoscale electrostatic microtubule disassembly forces between positively charged molecules in kinetochores and negative charges on plus ends of microtubules have been implicated in poleward chromosome motions and may also contribute to antipoleward chromosome movements. We propose that chromosome congression can be understood in terms of antipoleward nanoscale electrostatic microtubule assembly forces between negatively charged microtubule plus ends and like-charged chromosome arms, acting in conjunction with poleward microtubule disassembly forces. Several other aspects of post-attachment prometaphase chromosome motions, as well as metaphase oscillations, are consistently explained within this framework.

## Introduction

Poleward and antipoleward chromosome movements occur intermittently during prometaphase and metaphase. Poleward motion dominates during anaphase-A, while antipoleward motions dominate during the congressional movement of chromosomes to the cell equator. The apparent complexity of these motions has challenged scientific explanation for over a hundred years.

Experiments have shown that during prometaphase each pair of sister chromatids attaches by a kinetochore to the outside wall of a single microtubule, resulting in a rapid microtubule sliding movement toward a pole [1]. This motion is generally thought to be driven by molecular motors; specifically, the speed (20–50 μm per minute) of kinetochores along microtubule walls is consistent with known molecular motor behavior [1]. Current thought on chromosome motility, however, does not appear to favor molecular motors for post-attachment force generation.

As discussed elsewhere [2-5], force generation by nanoscale electrostatic microtubule disassembly forces between positive charges at kinetochores and negative charges on microtubule plus ends may be responsible for chromosome poleward motility during mitosis. We propose here that antipoleward nanoscale electrostatic microtubule assembly forces acting at chromosome arms combined with poleward forces are responsible for chromosome congression, and that this combination is consistent with other post-attachment prometaphase motions as well as metaphase chromosome oscillations.

The approach of kinetochores to the poles result in their movement to within critical distances of the ends of other (*astral*) microtubules emanating from the closer pole. Importantly, electrostatic forces increase significantly between charged surfaces separated by 3 nm or less (see below). The resulting proximity--in conjunction with (1) an electrostatic attraction between positively charged kinetochores and negatively charged ends of astral microtubules, (2) an electrostatic repulsion between negatively charged chromosome arms in the chromatid pair and neighboring negatively charged astral microtubule ends, and (3) constant thermal agitation—is likely integral to the orientation and end-on attachment of kinetochores to free microtubule plus ends [2]. Following a *monovalent* (or *mono-oriented*) attachment to one pole, chromosomes subsequently move at considerably slower speeds (a few μm per minute) throughout prometaphase [6]. In particular, a period of slow movements toward and away from a pole ensues until close proximity of the free microtubule end with the other (*sister*) kinetochore in the chromatid pair results in a *bivalent* (*bioriented*) attachment. Attachments of additional microtubules from both poles follow. After a sister kinetochore becomes attached to microtubules from the opposite pole, the chromosomes perform a slow (1–2 μm per minute) congressional motion to the spindle equator, resulting in the well-known metaphase alignment of chromatid pairs [6].

## Antipoleward nanoscale electrostatic assembly force

The permittivity (kϵ_o_) of the first few water layers outside a charged surface is an order of magnitude smaller than that of the bulk phase [7], and the effective permittivity of water as a function of distance from a charged surface increases monotonically from 4–6 ϵ_o_ at the interface to 78 ϵ_o_ at a distance of 25 nm from the interface. The values of the dielectric constants k (x) at distances (x) of 1, 2, 3, and 4 nm from a charged surface are 9, 21, 40, and 60, respectively [8]. Additionally, layered water adhering to charged molecules greatly reduces counterion (*Debye*) screening for small distances from their surfaces. Such water layering to charged proteins (e.g., microtubules) has long been theorized [9,10], and confirmed experimentally [11].

The interpolated values of k(x) for separations between charged surfaces of up to 3 nm are 5, 9, 9 and 5 for x = 0, 1, 2 and 3 respectively, where the charged surfaces are at x = 0 and x = 3 nm (the experimental value of k(x) at both x = 0 and x = 3 is 5, and symmetry and the experimental numbers dictate the values of 9 in between). The distance range of 1–3 nm between charged molecular surfaces is appropriate because 1 nm may be taken as the thickness of layered water adsorbed to each charged surface [10,12], and for charged molecular surface separations up to 3 nm, counterion screening would be virtually eliminated. Thus electrostatic force is increased over the distances allowed by reduced Debye screening, and is further increased (by an order of magnitude) due to an order of magnitude reduction in the dielectric constant between the charged surfaces. For brevity, separations of up to 3 nm (and -- due to the reduced dielectric constant between charged molecular surfaces -- 1 to 2 nm beyond) between charged surfaces will hereafter be designated as *critical distances/gaps*.

**Figure 1:**
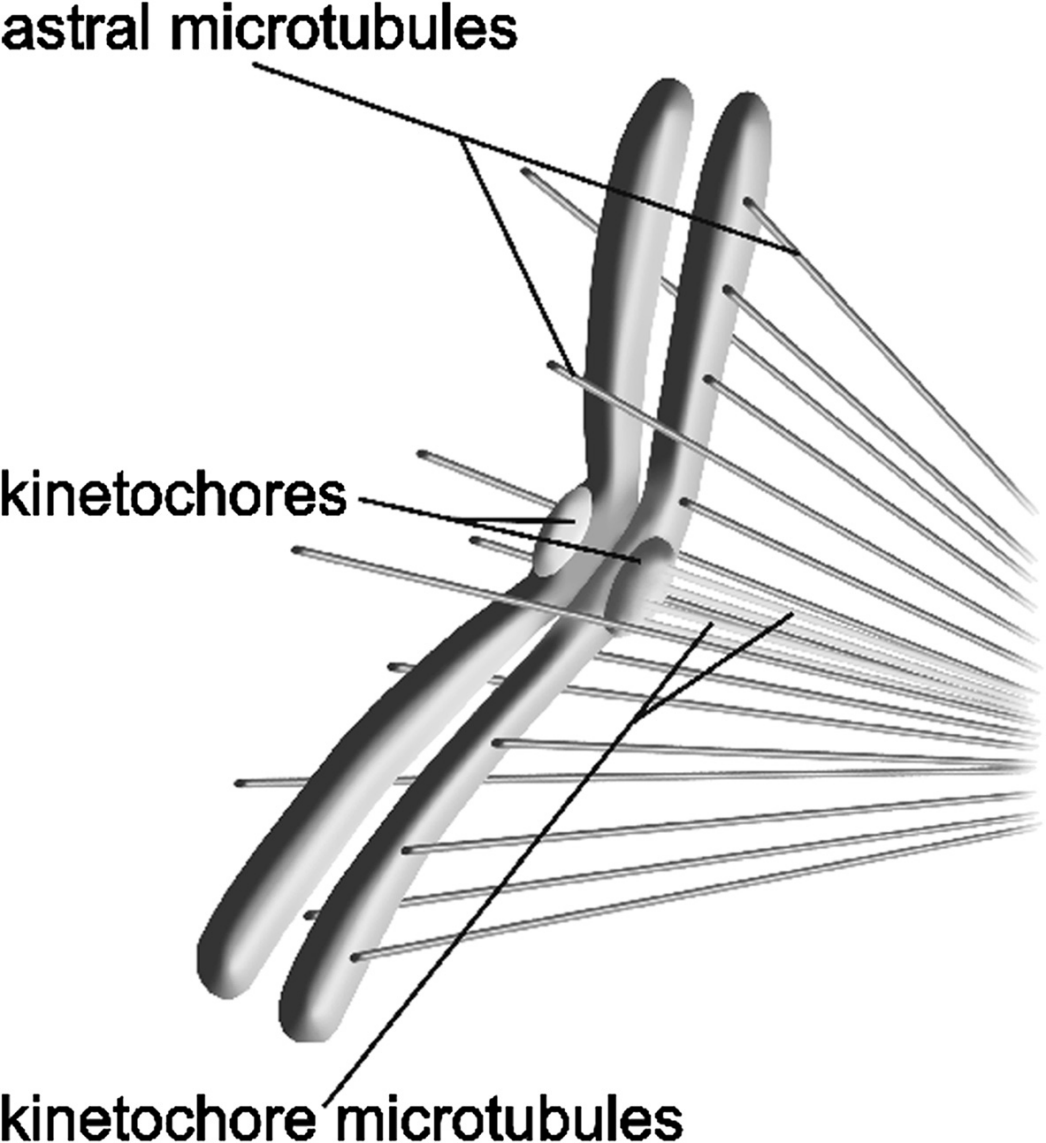
**Antipoleward electrostatic force between microtubules and chromosome arms.** An antipoleward force results from electrostatic repulsion between negatively charged plus ends of microtubules and negatively charged chromosome arms.

Due to the strong negative charge carried by chromosome arms, they are repelled from negatively charged free ends of astral microtubules in the polar region. Microtubule polymerization occurs in relatively large gaps that result from smaller (near critical) gap-dependant electrostatic repulsion between negatively charged microtubule plus ends and negatively charged chromosome arms. This process continues with other constantly changing subsets of smaller and larger gaps, causing chromosomes to be continuously repelled from poles. This mechanism may account for the antipoleward *astral exclusion force,* or *polar wind*, the precise nature of which has been sought since it was first observed [13]. The interaction between astral microtubules and chromosome arms is depicted in Figure 1.

As a chromatid pair moves farther from a pole, electrostatic repulsive forces between the negatively charged free ends of astral microtubules and chromosomes decreases as the microtubules fan radially outward. At a surface defined by the microtubule ends, the charge density and therefore the force, will decrease according to an “inverse square law”. Specifically, the repulsive force on a chromosome arm depends on the total number N of negatively charged microtubule free ends from which it is repelled; thus F ~ N q, where q is the charge at the end of a microtubule. For N microtubules fanning radially outward from a pole, the total charge N q is distributed over an area that increases as the distance r from the pole squared (r^2^), and the effective charge per unit area at a surface defined by the microtubule ends decreases as the inverse of the distance squared (1/r^2^). This results in a nanoscale repulsive electrostatic antipoleward force on chromosome arms that decreases with an *inverse square* (1/r^2^) dependence on the polar distance.

**Figure 2:**
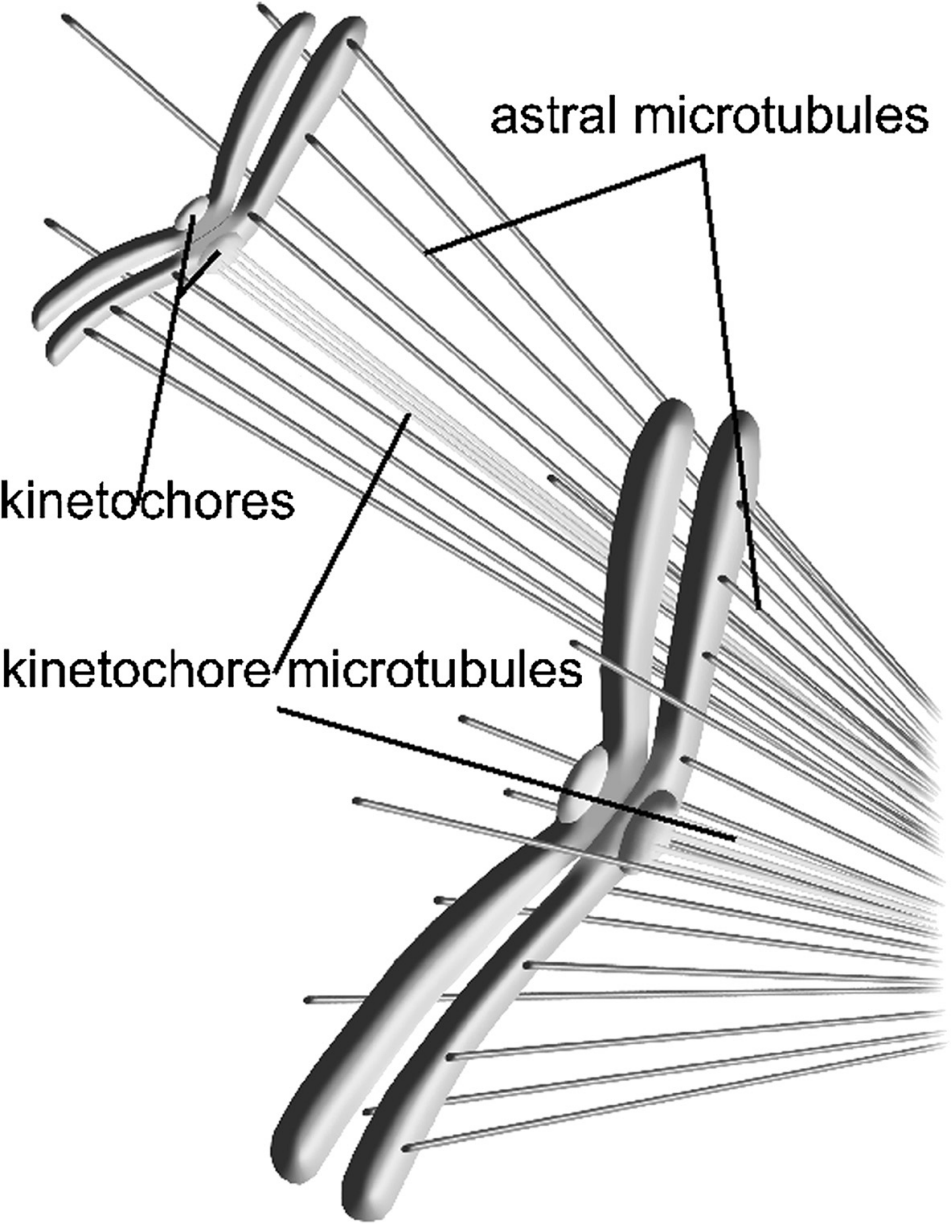
**Robust antipoleward inverse square repulsive force.** Two chromatid pairs at differing polar distances are depicted showing the robust inverse square dependence of the nanoscale repulsive electrostatic antipoleward force.

The falloff is even more pronounced than an inverse square dependence predicts due to the decreased number of microtubule free ends more distant from the poles, thus further reducing the antipoleward force (Figure 2). We refer to this effect as the *robust inverse square antipoleward force.*

## Prometaphase and metaphase chromosome motions

Microtubule polymerization and depolymerization, in combination with poleward and antipoleward forces, are sufficient to account for the observed motion of monovalently attached chromosomes. Due to fluctuations in both the number of disassembling kinetochore microtubules (interacting with kinetochores and centrosomes), and in the number of assembling astral microtubules (responsible for the antipoleward force acting at chromosome arms), these opposing forces result in a “tug of war” consistent with experimentally observed movements toward and away from a pole for a monovalently attached chromatid pair [6].

After a bivalent attachment is established, the attractive force to the distal pole opposes the attractive force to the proximal pole. The robust inverse square astral exclusion force results in greater repulsion from the proximal pole, and combined with a growing number of kinetochore attachments to microtubules from the distal pole (tending to equalize poleward disassembly forces), generates a relatively sustained congressional motion away from the proximal pole, as observed experimentally [6]. We emphasize that—within the context of our model--the dominance of the robust inverse square antipoleward force from the proximal pole is primarily responsible for chromosome congression. While some studies suggest that chromokinesins are involved in generating antipoleward force, others indicate that they are not essential for chromosome congression [14]. Note also that microtubule assembly at kinetochores and poles can occur; however, because the necessary inverse square dependence of the antipoleward microtubule assembly force cannot be derived from microtubule assembly at kinetochores or spindle poles, it is likely that assembly at either location is in passive stochastic response to assembly at chromosome arms, or to tension caused by poleward forces on sister kinetochores.

As a chromatid pair congresses to the midcell region, the number of attachments to both poles will tend to be the same as will the number of microtubules interacting with chromosome arms, and thus equilibrium of poleward directed forces and antipoleward astral exclusion forces will be approached. Without specifying their exact nature, such balanced pairs of attractive and repulsive forces have previously been postulated for the metaphase alignment of chromatid pairs [15].

An explanation of experimentally observed mid-cell metaphase oscillations just prior to anaphase-A provides an example of the predictability and minimal assumptions nature of the model. In agreement with experimental observations [16], our model predicts that the poleward force on a chromosome from kinetochore microtubule disassembly (at kinetochores and poles) depends on the total number of kinetochore microtubules. At the metaphase “plate”, the bivalent attachment of chromatid pairs ensures that the poleward-directed electrostatic disassembly force on one chromatid at a given moment could be greater than that of the sister chromatid’s kinetochore attached to the opposite pole. An imbalance of these poleward forces results from statistical fluctuations in the number of force generating kinetochore microtubules. This situation, coupled with similar fluctuations in the number of astral microtubules responsible for the antipoleward astral exclusion force on a chromatid pair, can result in a momentary motion toward a pole in the direction of the instantaneous net force. However, due to the robust inverse square dependence of the repulsive astral exclusion force and the approximate equality of poleward-directed microtubule disassembly forces for chromatid pairs in the midcell region, the greater force of repulsion from the proximal pole will eventually reverse the direction of motion resulting in midcell metaphase oscillations, as observed experimentally [6].

Midcell metaphase oscillations are indirect evidence for a continuing increase in the disassembly to assembly probability ratio resulting in parity for microtubule assembly and disassembly probabilities. As discussed elsewhere [17], this continuing increase in the microtubule disassembly/assembly ratio may be due to a continuously decreasing intracellular pH (pHi). At late metaphase, before anaphase-A, experiments reveal that poleward motions of sister kinetochores stretch the intervening centromeric chromatin producing high kinetochore tensions, most likely caused by a continuing disassembly to assembly probability ratio increase. At these high tensions, microtubule plus ends often switch from a depolymerization to a polymerization state of dynamic instability. This may be explained by kinetochore microtubule free ends taking up the slack by polymerization to sustain attachment and resist further centromeric chromatin stretching, collectively known as the “slip-clutch mechanism” [18,19], as explained below.

Microtubule assembly at a kinetochore or a pole is regarded here as operating in passive response to (1) the robust inverse square electrostatic antipoleward force acting between the plus ends of microtubules and chromosome arms and/or (2) an electrostatic microtubule disassembly force at a sister kinetochore or at poles. At the highest tensions, electrostatic forces acting over critical distances between protofilament free ends and kinetochores are effective in maintaining coupling while larger protofilament gaps in the same or other microtubules are passively filled in. Additionally, the robust inverse square microtubule assembly force acting at a sister chromatid’s arms provides a feedback mechanism to resist detachment. This explanation of the slip-clutch mechanism follows as a direct consequence of the proposed model with no additional assumptions. As discussed elsewhere [17], anaphase-A results from the eventual complete domination of microtubule disassembly over assembly, resulting in a poleward disassembly force that dwarfs the antipoleward microtubule assembly force.

## Conclusions

Both the range and strength of electrostatic forces within cells is greater than counterion screening would dictate. Chromosome congression likely results from a combination of poleward forces with a robust inverse square antipoleward electrostatic microtubule assembly force acting at chromosome arms. The dominance of the inverse square dependence of the antipoleward microtubule assembly force over the poleward microtubule dissasembly force is primarily responsible for chromosome congression as well as metaphase chromosome oscillations. Chromosome end-on attachment orientation and the “slip-clutch” mechanism are consistent with this combination of opposing forces.

## Competing interests

The authors declare that they have no competing interests.

## Authors’ contributions

DHS made intellectual contributions and drafted the manuscript. LJG conceived the study. All authors read and approved the final manuscript.

## References

[B1] Rieder CL Alexander SP Kinetochores are transported poleward along a single astral microtubule during chromosomes attachment to the spindle in Newt lung cells J Cell Biol 1990 110 81 95 10.1083/jcb.110.1.81 2295685 PMC2115982

[B2] Gagliardi LJ Electrostatic force in prometaphase, metaphase, and anaphase-A chromosome motions Phys Rev E 2002 66 011901–1 011901-8 10.1103/PhysRevE.66.011901 12241378

[B3] Gagliardi LJ Electrostatic force generation in chromosome motions during mitosis J Electrostat 2005 63 309 327 10.1016/j.elstat.2004.09.007

[B4] Guimaraes GJ Dong Y McEwen BF DeLuca JG Kinetochore microtubule attachment relies on the disordered N-terminal tail domain of Hec1 Current Biol 2008 18 1778 1784 10.1016/j.cub.2008.08.012 PMC2753282 19026543

[B5] Miller SA Johnson ML Stukenberg PT Kinetochore attachments require an interaction between unstructured tails on microtubules and Ndc80/Hec1 Current Biol 2008 18 1785 1791 10.1016/j.cub.2008.11.007 PMC3145211 19026542

[B6] Inoue S Salmon ED Force generation by microtubule assembly/disassembly in mitosis and related movements Mol Biol Cell 1995 6 1619 1640 10.1091/mbc.6.12.1619 8590794 PMC301321

[B7] Bockris JO Reddy AKN Modern Electrochemistry 1977 New York: Plenum Press

[B8] Teschke O Ceotto G de Souza EF Interfacial water dielectric permittivity profile measurements using atomic force microscopy Phys Rev E 2001 64 011605–1 011605–10 10.1103/PhysRevE.64.011605 11461268

[B9] Jordan-Lloyd D Shore A The Chemistry of Proteins 1938 London: J. A. Churchill Publishing Company

[B10] Pauling L The adsorption of water by proteins J Am Chem Soc 1945 67 555 557 10.1021/ja01220a017

[B11] Toney MF Howard JN Richer J Borges GL Gordon JG Melroy OR Wiesler DG Yee D Sorensen L Voltage-dependent ordering of water molecules at an electrode-electrolyte interface Nature 1994 368 444 446 10.1038/368444a0

[B12] Pollack GH Cells, Gels and the Engines of Life 2001 Seattle: Ebner and Sons Publishers 69

[B13] Rieder CL Davison EA Jensen LCW Oscillatory movements of mono oriented chromosomes and their position relative to the spindle pole result from the ejection properties of the aster and half-spindle J Cell Biol 1986 103 581 591 10.1083/jcb.103.2.581 3733881 PMC2113830

[B14] Levesque AA Compton DA The chromokinesin Kid is necessary for chromosome arm orientation and oscillation, but not congression, on mitotic spindles J Cell Biol 2001 154 6 1135 46 10.1083/jcb.200106093 11564754 PMC2150818

[B15] Alberts B Bray D Lewis J Raff M Roberts MK Watson JD Molecular Biology of the Cell 1994 New York: Garland Publishing Company 926

[B16] Hays TS Salmon ED Poleward force at kinetochores in metaphase depends on the number of kinetochore microtubules J Cell Biol 1990 110 391 404 10.1083/jcb.110.2.391 2298810 PMC2116015

[B17] Gagliardi LJ Shain DH Is intracellular pH a clock for mitosis? Theor Biol Med Model 2013 10 8 10.1186/1742-4682-10-8 23402214 PMC3583677

[B18] Rieder CL Salmon ED Motile kinetochores and polar ejection forces dictate chromosome position on the vertebrate mitotic spindle J Cell Biol 1994 124 223 233 10.1083/jcb.124.3.223 8294508 PMC2119939

[B19] Maddox P Straight A Coughlin P Michison TJ Salmon ED Direct observation of microtubule dynamics at kinetochores in Xenopus extract spindles: implications for spindle mechanics J Cell Biol 2003 162 377 382 10.1083/jcb.200301088 12900391 PMC2172681

